# A gestational vulnerability window for smoking exposure and the increased risk of preterm birth: how timing and intensity of maternal smoking matter

**DOI:** 10.1186/s12978-019-0705-x

**Published:** 2019-04-16

**Authors:** Anthony J. Kondracki, Sandra L. Hofferth

**Affiliations:** School of Public Health, Department of Family Science, University of Maryland, 4200 Valley Drive, College Park, MD 20742 USA

**Keywords:** Preterm birth, Trimester, Pregnancy, Timing of smoking, Cessation

## Abstract

**Background:**

Reducing the incidence of preterm birth is a national priority. Maternal cigarette smoking is strongly and consistently associated with preterm birth. The objective of this study was to examine prenatal exposure based on combined measures of timing (by trimester) and intensity level (the number of cigarettes smoked per day) of maternal smoking to identify a pregnancy period with the highest risk of preterm birth.

**Methods:**

A sample of 2,485,743 singleton births was drawn from the 2010 National Center of Health Statistics (NCHS) linked birth/infant death file of US residents in 33 states that implemented the revised 2003 birth certificate. Nine mutually exclusive smoking status categories were created to assess prenatal exposure across pregnancy in association with preterm birth. Gestational age was based on the obstetric estimate. Multiple logistic regression analyses were conducted to compare the odds of preterm birth among women who smoked at different intensity levels in the second or third trimester with those who smoked only in the first trimester.

**Results:**

Overall, 7.95% of women had a preterm birth; 8.90% of low intensity (less than a pack/day) smokers in the first trimester only, 12.99% of low and 15.38% of high intensity (pack a day or more) smokers in the first two trimesters, and 10.56% of low and 11.35% of high intensity smokers in all three trimesters delivered preterm. First and second trimester high (aOR 1.85, 95% CI: 1.66, 2.06) and low intensity smokers (aOR 1.51, 95% CI: 1.41, 1.61) had higher odds of preterm birth compared to those who smoked less than a pack a day only in the first trimester, but the odds did not increase for all three trimester smokers relative to the first and second trimester smokers. In sensitivity analysis, adjustment for exposure misclassification error corrected data and testing for effect modification by maternal race/ethnicity found no significant interaction.

**Conclusions:**

This study documented a biologically plausible vulnerability window for smoking exposure and the increased risk of preterm birth. For women who do not modify their smoking behavior preconception, preterm birth risk of smoking remains low until late in the first trimester.

## Plain English summary

Preterm birth before 37 weeks gestation occurs in about 5 to 18% of all pregnancies and is associated with increased morbidity and mortality in newborns. Smoking during pregnancy is a potentially preventable risk factor for preterm birth, yet nearly one-third of reproductive age women (18–44 years old) are daily smokers and at least half of women continue smoking during pregnancy. Infants whose mothers do not smoke at all have dramatically better outcomes. Although women try to quit smoking upon pregnancy recognition, little is known about when smoking cessation during pregnancy matters most for preterm birth. To date, few studies thoroughly examined variability in patterns of timing and intensity of smoking to capture the extent of prenatal exposure across trimesters of pregnancy. This is the first population-based study to utilize combined measures of timing, intensity and duration of maternal smoking to identify a potential biologically plausible window of enhanced vulnerability to smoking exposure, which is late in the first trimester, to avoid preterm birth.

## Background

Preterm birth of less than 37 weeks gestation occurs in about 5 to 18% of pregnancies. [1] Reducing the incidence of preterm birth by 10% is a key objective of Healthy People 2020. [2]

In the United States, the overall rate of preterm birth rose from 9.63% in 2015 to 9.85% in 2016 and to 9.93% in 2017, and for singleton births only the rate increased from 7.82% in 2015 to 8.02% in 2016 and to 8.13% in 2017. [3] Racial and ethnic disparities exist such that in 2017 non-Hispanic Black (13.93%) and Hispanic mothers (9.62%) had a higher rate of preterm birth than non-Hispanic White (9.05%) or Asian mothers (8.53%). [3] About 5% of preterm births are extremely preterm (20 0/7 to 27 6/7 weeks gestation), 15% severely preterm (28 0/7 to 31 6/7 weeks), 20% moderately preterm (32 0/7 to 33 6/7 weeks), and the majority (60%) and the fastest growing group are late preterm births (34 0/7 to 36 6/7 weeks gestation). [4] In the US in 2010, two-thirds (66.7%) of all infant deaths were attributed to the 12% of infants born preterm. [5] The mortality rate for infants born very preterm was 165.57 per 1000 of live births, which was 74 times the rate of 2.25 for term infants, and for moderately preterm infants the rate was 15.83, which was seven times the rate for term infants. [5] Shortened gestational age indicates newborn immaturity and predicts poor health at birth and during the life course. [6] Estimated health care costs related to preterm delivery, reported in 2005, were at least $26 billion annually ($51,600 per infant). [6]

Approximately one-third of reproductive age women (18–44) in the US are daily cigarette smokers. [7] Tobacco smoke contains more than 4000 harmful chemicals among which nicotine is the most addictive substance and a reproductive toxicant. [8] Maternal smoking has been recognized as an independent risk factor for preterm birth, fetal growth restriction and low birthweight, [9–11] and has been associated with miscarriage, [12] placenta praevia and placental abruption. [13] Health care expenses for smoking mothers and their newborns can be 66% higher than for nonsmoking mothers. [14]

National reports indicate that smoking in pregnancy is more prevalent among non-Hispanic White women, 20–24 years old, and less educated women. [7] Among the 10.9% of women who smoked in the 3 months prior to pregnancy, 24.2% quit smoking before becoming pregnant and 8.4% of women smoked at any time during pregnancy. [7] The prevalence of smoking was 8.2% in the first trimester, 7.0% in the second and 6.6% in the third trimester. [7] On average, one fifth of smoking pregnant women quit spontaneously at the time of their first prenatal visit, but 70% of those who quit, returned to smoking within six months after childbirth. [15, 16] Women 18–24 years old were most likely to have attempted to quit (68.4%), but least likely to have quit smoking (26.3%) in pregnancy. [15]

It is well documented that infants whose mothers do not smoke at all have dramatically better outcomes, but little is known whether and when during pregnancy smoking cessation matters for preterm birth. Prior studies linking evidence of smoking any time during pregnancy with the increased risk of adverse birth outcomes suggested that quitting early in pregnancy reduced [9, 17] or reversed such risk. [18] Based on an 11-state US subpopulation analysis, quitting in the first trimester lowered the risk of delivering preterm by 31%, to the level of nonsmokers, whereas smoking into the second trimester and then quitting did not have the same effect. [19] Some researchers questioned whether the number of cigarettes smoked increases the risk of delivering a growth restricted and low birthweight infant [9, 10, 20] and how it influences the risk of both overall and spontaneous preterm birth. [17] Smoking more than ten cigarettes per day has been significantly linked to preterm delivery [17, 20] and a greater number of cigarettes smoked, irrespective of trimester of pregnancy, is likely to increase the risk. [20]

Given low rates of quitting and high rates of relapse among women who smoke in the first trimester of pregnancy, [15, 16] we seek to understand the implications of prenatal exposure from continued smoking or quitting early or later in pregnancy. Associations with preterm birth were most commonly reported for maternal smoking in early pregnancy (the first trimester) [19] or mid-trimester; [21] however, a specific time by which smoking in pregnancy must stop to prevent preterm delivery is unclear. To examine prenatal exposure status more thoroughly in association with the increased risk of preterm birth, we combined measures of timing (per trimester), intensity (by the number of cigarettes smoked per day) and duration (consistency) of maternal smoking into nine mutually exclusive smoking status categories. We hypothesize that systematically comparing various smoking patterns across trimesters of pregnancy can establish smoking exposure and help identify biologically plausible window(s) for enhanced vulnerability to preterm birth.

## Methods

### Data source

As a data source in this study, we used the electronic 2010 National Center for Health Statistics (NCHS) linked birth/infant death dataset (*N* = 4,007,105) of all births registered in the 33 states that had implemented the 2003 revised version of the birth certificate, representing 76% of all US births. [5] The NCHS in cooperation with the National Vital Statistics System (NVSS) include birth certificate data in the natality file allowing public access to deidentified information. [22] During the twentieth century, the birth certificate had 11 revisions to improve “completeness and accuracy” of data collection and reporting, [24] and the last 2003 revised version gradually replaced the previous 1989 version across the country. [23] Availability of more detailed information on the timing and intensity of smoking during pregnancy in the 2003 revised version made this study possible. [24]

### Study population and study design

This cross-sectional study included the entire population of 2,485,743 singleton live births in the United States in 2010, after excluding multiple births (*n* = 138,264), births in states with unrevised certificates (*n* = 906,068), births with missing data on maternal race (*n* = 2146) and on other maternal demographic characteristics (*n* = 10,214), and on smoking by trimester (*n* = 465,084).

### Definition and measurement

Pregnancy was defined by three trimesters (gestational stages): the first trimester (months 1–3- or 1–13-weeks gestation), the second trimester (months 4–6 or 14–27 weeks gestation), and the third trimester (months 7–9 or 28 weeks to delivery). [25]

Preterm birth was defined as delivering a singleton newborn between 20 and less than 37 weeks gestation, based on the best obstetric estimate (OE) of gestation at birth that combines date of maternal last menstrual period (LMP) and ultrasound (US) measurement available in the revised birth certificate. [26] The OE has more validity than the LMP-based estimate alone, because recall error is minimized and births are less likely to be misclassified. [27]

A vulnerability window for prenatal smoking exposure was defined as the most sensitive period (trimester) in pregnancy [28, 29], as indicated by the increased odds of preterm birth.

### Exposure variable

The exposure variable was cigarette smoking during pregnancy. On the revised birth certificate the mother reports the average number of cigarettes smoked per day in each trimester of pregnancy and before pregnancy. [7] In this study, timing of cigarette smoking was measured by trimester and intensity level was classified as low intensity (1–19 cigarettes/day or less than a pack/day) and high intensity (≥20 cigarettes/day or a pack/day or more), based on prior research. [20]

### Outcome variable

The outcome variable was preterm birth defined categorically based on gestational age of less than 37 weeks gestation or between 20 0/7 and 36 6/7 weeks gestation.

### Covariates

We combined race and ethnicity to form five categories: non-Hispanic White, non-Hispanic Black, Hispanic, non-Hispanic Asian, and American Indian. Maternal age categories were 12–19, 20–34 and 35 years old or older; maternal education was categorized as less than high school, high school graduate, some college or associate degree, and a bachelor’s degree or higher; and marital status was married or unmarried.

### Exposure status

Prenatal exposure status was examined based on variability in maternal smoking patterns across trimesters. [30–32] Women who quit smoking before pregnancy appeared to have a rate of exposure similar to non-smoking women [21] and we did not study them separately. We assumed that women who reported smoking were active smokers because nonsmokers would be less likely to report active smoking in pregnancy. [33, 34] We classified women as non-smoking only if they reported no cigarette use during any trimester. Consistent ever-smokers could have smoked in the first trimester only, in the first and second trimesters only, or in all three trimesters. A small proportion of women were inconsistent smokers who reported smoking in the first and in the third trimester only and denied smoking in the second trimester, who reported smoking in the second trimester only or in the second and the third trimester, or who smoked only in the third trimester.

Our study advanced standard methodology by creating nine mutually exclusive smoking intensity status categories combining measures of timing (trimesters in which smoking was reported), intensity (the number of cigarettes smoked per day) and duration (consistency across trimesters) of smoking to thoroughly assess distribution of prenatal exposure across trimesters: low-intensity smokers (1–19 cigarettes/day or less than a pack/day) in the first but not in the second trimester (reference group); high-intensity smokers (≥20 cigarettes/day or a pack/day or more) in the first but not in the second trimester; low-intensity smokers in the first and second trimesters; high-intensity smokers in the first and second trimesters; low-intensity smokers who started smoking in the second or third trimesters (did not smoke in the first trimester); high-intensity smokers who started smoking in the second or third trimesters; low-intensity smokers in all three trimesters; high-intensity smokers in all three trimesters; and nonsmokers (no trimester smokers).

### Quality of self-reported smoking data

Almost 25% of pregnant women do not disclose smoking throughout pregnancy, related in part to social stigma [36–38]. Information on smoking during pregnancy collected from mothers on birth certificates showed moderate to high agreement when compared to other self-reported information from the Pregnancy Risk Assessment Monitoring System (PRAMS), and slightly less agreement when compared with medical records. [36, 37] Third trimester prenatal smoking reported on the birth certificate was highly correlated with newborn cotinine levels. [35] Cotinine is a primary product of nicotine metabolism detected in blood, saliva or urine, and is considered a biochemical marker (biomarker) and gold standard measure of choice to validate current smoking status. [39, 40] While prior prospective epidemiological studies showed cotinine levels to be correlated with self-reported number of cigarettes smoked at any given time in pregnancy, the results have been inconclusive among self-reported quitters. [27, 31, 41, 42]

### Statistical analyses

In this study, the entire population of 2,485,743 singleton live births in the US in 2010 was separated into two gestational age subgroups: births between 20 weeks gestation and term (second and third trimester births) and births between 28 weeks gestation and term (third trimester births only). Conceptually, we first estimated preterm birth (20- < 37 weeks gestation) rates for these two gestational age subgroups across maternal race/ethnicity, age, educational attainment and marital status (Table 1), and performed similar analyses according to maternal smoking status categories (Table 2). Next, we conducted multiple logistic regression analyses to assess crude (without adjustment for confounding) and adjusted (with adjustment for confounding) odds ratios of preterm births and 95% confidence intervals (Table 3). To identify a potential vulnerability window during which prenatal smoking exposure exerts greatest effect on the increased risk of preterm birth, we sought a combination of trimester-timing and intensity-level maternal smoking that was most strongly linked to the risk of preterm birth. Our focus was on comparing prenatal exposure by trimester from the beginning of pregnancy and we assumed that women lower their smoking intensity and quit thereafter, thus low intensity smoking in the first trimester only was the reference category. [41] The main comparison involved women who smoked in the first and in the second trimester versus in the first trimester (referent), and those who smoked in the first, the second, and third trimester versus in the first trimester (referent). In order to determine which trimester smoking exposure was associated with the highest risk of preterm birth, we also examined inconsistent smokers. Significance level was set at *p* = 0.05.

**Table 1:** Percentage of all preterm births (20- < 37 weeks gestation) and third trimester preterm births (28- < 37 weeks gestation) across maternal characteristics, singleton births, United States, 2010

	N (%)	Preterm (%) 20- < 37 weeks	Preterm (%) 28- < 37 weeks
Total N (%)	2,485,743 (100)	(7.95)	(7.49)
Race/ethnicity			
Non-Hispanic White	1,320,489 (53.12)	(7.27)	(6.94)
Non-Hispanic Black	296,389 (11.92)	(11.48)	(10.37)
Hispanic	690,911 (27.79)	(7.84)	(7.40)
Non-Hispanic Asian	154,607 (6.22)	(7.29)	(6.96)
Non-Hispanic American Indian	24,992 (0.84)	(9.30)	(8.87)
Age (years)			
12–19	237,060 (9.54)	(9.08)	(8.45)
20–34	1,896,901 (76.31)	(7.62)	(7.18)
35 or older	351,782 (14.15)	(8.99)	(8.50)
Education			
Less than high school	506,474 (20.38)	(8.94)	(8.39)
High school	618,388 (24.88)	(8.62)	(8.06)
Some college	691,508 (27.82)	(8.26)	(7.78)
Graduate school	669,373 (26.93)	(6.28)	(5.99)
Marital Status			
Unmarried	1,001,617 (40.29)	(9.22)	(8.58)
Married	1,484,126 (59.71)	(7.10)	(6.76)

**Table 2:** Percentage of all preterm births (20- < 37 weeks gestation) and third trimester preterm births (28- < 37 weeks gestation) by maternal smoking status, singleton births United States, 2010

Maternal Smoking Status	N (%)	Preterm (%) 20- < 37 weeks	Preterm (%) 28- < 37 weeks
No trimester smokers	2,253,887 (90.67)	(7.68)	(7.23)
1st trimester only, low	28,560 (1.15)	(8.90)	(8.14)
1st trimester only, high	4909 (0.20)	(8.68)	(7.87)
1st & 2nd trimesters, low	11,619 (0.47)	(12.99)	(10.58)
1st & 2nd trimesters, high	2835 (0.11)	(15.38)	(12.57)
2nd & 3rd trimesters, low	5759 (0.23)	(8.73)	(8.34)
2nd& 3rd trimesters, high	584 (0.02)	(9.42)	(8.79)
All 3 trimesters, low	130,060 (5.23)	(10.56)	(10.09)
All 3 trimesters, high	47,530 (1.91)	(11.35)	(10.99)

Low intensity: 1–19 cigarettes/day; high intensity: ≥ 20 cigarettes/day

**Table 3:** The odds of all preterm births (20- < 37 weeks gestation) and third trimester preterm births (28- < 37 weeks gestation) across maternal smoking status categories

Preterm 20- < 37 weeks				
Smoking Status	Crude OR	95% CI	Adjusted OR^a^	95% CI
No trimester smokers	0.85	0.82, 0.89*	0.90	0.86, 0.94*
1st trimester only, low (ref.)	1	1	1	1
1st trimester only, high	0.97	0.87, 1.08	0.98	0.88, 1.09
1st & 2nd trimesters, low	1.53	1.43, 1.64*	1.51	1.41, 1.61*
1st & 2nd trimesters, high	1.86	1.67, 2.07*	1.85	1.66, 2.06*
2nd & 3rd trimesters, low	0.98	0.89, 1.08	0.95	0.86, 1.05
2nd & 3rd trimesters, high	1.06	0.80, 1.41	1.03	0.77, 1.36
All 3 trimesters, low	1.21	1.16, 1.26*	1.20	1.14, 1.25*
All 3 trimesters, high	1.31	1.25, 1.38*	1.33	1.26, 1.40*
Preterm 28- < 37 weeks				
Smoking Status	Crude OR	95% CI	Adjusted OR^a^	95% CI
No trimester smokers	0.88	0.84, 0.92*	0.93	0.89, 0.97*
1st trimester only, low (ref.)	1	1	1	1
1st trimester only, high	0.96	0.86, 1.08	0.97	0.86, 1.08
1st & 2nd trimesters, low	1.34	1.24, 1.44*	1.32	1.22, 1.42*
1st & 2nd trimesters, high	1.62	1.44, 1.83*	1.61	1.43, 1.82*
2nd & 3rd trimesters, low	1.03	0.93, 1.14	1.00	0.90, 1.11
2nd & 3rd trimesters, high	1.09	0.81, 1.46	1.05	0.79, 1.40
All 3 trimesters, low	1.27	1.21, 1.33*	1.25	1.20, 1.31*
All 3 trimesters, high	1.39	1.32, 1.47*	1.40	1.33, 1.48*

^a^Analyses were adjusted for: maternal race/ethnicity, age, education, and marital status; low intensity: 1–19 cigarettes/day; high intensity: ≥ 20 cigarettes/day; *p < 0.05; CI-confidence interval

Additionally, we conducted a sensitivity analysis for potential exposure misclassification error based on maternal smoking self-reports by analyzing only those mothers who delivered in the third trimester, i.e., the second gestational age subgroup [44, 45] and separately tested for effect modification by maternal race/ethnicity on the association between high intensity smoking status in the first and the second trimester and the risk of preterm birth. [43, 47] The study analysis included the entire population, so weights were not utilized. SAS version 9.4 (SAS Institute Inc., Cary, NC) was used in all statistical computations.

This was a secondary analysis of deidentified and publicly available data and our study received Institutional Review Board (IRB) exemption from the University of Maryland. The authors have no financial or other conflict of interest.

## Results

Nearly 7.95% of mothers had a preterm birth (20 - < 37 weeks gestation) by the most inclusive definition and 7.49% mothers delivered preterm in the third trimester (28 - < 37 weeks gestation) (Table 1; left- and right-hand panels, respectively). Over half (53.12%) of mothers were non-Hispanic White who had a preterm birth rate of 7.27 and 11.92% were non-Hispanic Black mothers with a preterm birth rate of 11.48%.

More than 90% of women abstained from smoking during pregnancy (Table 2). Among women who smoked consistently during all three trimesters (7.14%), 5.23% smoked less than a pack/day and 1.91% smoked a pack/day or more. Women who reported smoking less than a pack/day (low intensity smokers) in the first trimester only (1.15%) were the reference category. In the first gestational age subgroup analysis (20 weeks gestation to term) 7.68% of women who abstained from smoking during pregnancy had a preterm birth (Table 2; left hand panel). In comparison, among women who smoked during all three trimesters, 10.56% of low intensity smokers (less than a pack/day) and 11.35% of high intensity smokers (more than a pack/day) delivered preterm (Table 2; left hand panel). In the second gestational age subgroup (28 weeks gestation to term) the results were similar, but preterm birth rates were lower (Table 2, right hand panel).

Two logistic regression models display results of crude and adjusted odds ratios (Table 3). Mothers who abstained from smoking had about 10% lower adjusted odds ratios of preterm birth in the first gestational age subgroup (aOR 0.90, 95% CI: 0.86, 0.94; Table 3, upper panel) and 7% in the second gestational age subgroup analysis (aOR 0.93, 95% CI: 0.89, 0.97; Table 3, lower panel), compared to the first trimester low intensity smokers (referent). Comparison of timing of prenatal exposure across trimesters indicates that not smoking in the first trimester but smoking in the second and third trimesters does not increase the risk of preterm birth, compared to smoking at low intensity in the first trimester only. Women who smoked more than a pack/day only in the first trimester did not have significantly higher odds of preterm birth, compared to women who smoked less than a pack a day (referent). In contrast, women who smoked less than a pack a day in the first and second trimesters had 51% higher odds of preterm birth (aOR 1.51, 95% CI: 1.41, 1.61) than those who smoked less than a pack/day in the first trimester only (referent) (Table 3). Finally, women who smoked more than a pack a day in the first and second trimesters had 85% higher odds of preterm birth (aOR 1.85, 95% CI: 1.66, 2.06), compared to women who smoked less than a pack/day in the first trimester only (referent). This suggests that the late part of the first trimester is a potential vulnerability period during which smoking exerts its greatest effect on the risk of preterm birth.

Interestingly, smoking in all three trimesters at low intensity showed a somewhat reduced association (regression attenuation) with preterm birth in the first (aOR 1.20, 95% CI: 1.14, 1.25) gestational age group, (Table 3; upper panel) relative to first trimester low intensity smoking (referent). We attributed these findings to an exposure misclassification error from suboptimal maternal self-reports. [44, 45] Mothers who delivered in the second trimester could not have smoked in the third trimester [38] and were excluded (*n* = 12,405; 0.46%) from the second gestational subgroup (i.e. third trimester births). Restricting the sample to the second gestational age subgroup (aOR 1.25, 95% CI: 1.20, 1.31) (Table 3; lower panel) raises the association somewhat. Restricting the sample and changing the reference group to low intensity smoking in the first and second trimesters (Table 4, lower panel) allowed statistical comparison of the risk among three-trimester smokers to that of first and second trimester smokers. The crude and adjusted odds ratios of preterm birth no longer differed significantly for low or high intensity smokers in all three trimesters, compared to the two-trimester smokers (referent category) (Table 4, lower panel). The statistically increased risk of preterm birth conferred by consistent prenatal cigarette smoke exposure in the first and later trimesters compared to those who smoked in the first trimester only remained in this sensitivity analysis. [46] Testing for effect modification in the regression model by maternal race/ethnicity between high intensity smoking status in the first and second trimesters and the risk of preterm birth showed no significant interaction, which was consistent with a prior study. [47]

**Table 4:** The odds of all preterm births (20- < 37 weeks gestation) and third trimester preterm births (28- < 37 weeks) across maternal smoking status categories (alternate reference)

Preterm 20- < 37 weeks				
Smoking Status	Crude OR	95% CI	Adjusted OR^a^	95% CI
No trimester smokers	0.56	0.53, 0.59*	0.60	0.57, 0.63*
1st trimester only, low	0.65	0.61, 0.70*	0.66	0.62, 0.71*
1st trimester only, high	0.64	0.57, 0.71*	0.65	0.58, 0.73*
1st & 2nd trimesters, low (ref.)	1	1	1	1
1st & 2nd trimesters,high	1.22	1.09, 1.37*	1.23	1.09, 1.38*
2nd & 3rd trimesters, low	0.64	0.58, 0.71*	0.63	0.57, 0.70*
2nd & 3rd trimesters, high	0.70	0.53, 0.92*	0.68	0.51, 0.90*
All 3 trimesters, low	0.79	0.75, 0.84*	0.79	0.75, 0.84*
All 3 trimesters, high	0.86	0.81, 0.91*	0.88	0.83, 0.94*
Preterm 28- < 37 weeks				
Smoking Status	Crude OR	95% CI	Adjusted OR^a^	95% CI
No trimester smokers	0.66	0.62, 0.70*	0.70	0.66, 0.75*
1st trimester only, low	0.75	0.70, 0.81*	0.76	0.71, 0.82*
1st trimester only, high	0.72	0.64, 0.82*	0.73	0.65, 0.83*
1st & 2nd trimesters, low (ref.)	1	1	1	1
1st & 2nd trimesters, high	1.22	1.07, 1.38*	1.22	1.08, 1.39*
2nd & 3rd trimesters, low	0.77	0.69, 0.86*	0.76	0.68, 0.85*
2nd & 3rd trimesters, high	0.82	0.61, 1.09	0.80	0.59, 1.07
All 3 trimesters, low	0.95	0.89, 1.01	0.95	0.89, 1.01
All 3 trimesters, high	1.04	0.98, 1.12	1.07	0.99, 1.14

^a^Analyses were adjusted for: maternal race/ethnicity, age, education, and marital status; low intensity: 1–19 cigarettes/day; high intensity: ≥ 20 cigarettes/day; *p < 0.05; CI-confidence interval

## Discussion

Many women quit smoking during pregnancy to reduce risk of harm to the developing fetus but the rate of successful quit attempts is low. [36] Although understanding the implications of varied patterns of maternal smoking from the onset of pregnancy until the time of delivery would be informative to women and health professionals, research to date has been limited. [30, 48] A recent meta-analysis revealed that almost half of studies use a binary measure of maternal smoking, usually collected after delivery, and poorly define intensity levels. [49] The present study assessed how the odds of preterm birth vary according to measures of timing (per trimester), intensity (the number of cigarettes smoked per day) and duration (consistency) of smoking in pregnancy, using first trimester quitting as a reference category. Women who did not smoke during the first trimester had a risk of preterm birth similar to those who quit during that trimester. Women who smoked more than a pack/day in the first trimester only did not have significantly higher odds of preterm birth compared to women who smoked less than a pack a day in the first trimester only (referent). This suggests that there is a window of time wherein quitting keeps the risk of preterm birth to a level comparable to nonsmokers, and intensity does not affect this risk. In contrast, women who smoked less or more than a pack a day in the first and second trimesters had a significantly higher odds of preterm birth compared to the referent category. Additionally, those who did not report smoking in the first trimester but smoked later did not have higher risk of preterm birth. The period late in the first and early in the second trimester is a potential biologically plausible vulnerability window for the risk of preterm birth that has been proposed in prior research [19, 21] and this research suggests that it is most pronounced late in the first trimester.

There may be substantial placental risks at about weeks 12 and 13 that have not yet been fully identified. It is already known that during the first trimester nicotine can interfere with hypoxic embryogenesis, placentation and organogenesis by generating excess reactive oxygen species resulting in direct oxidative damage to the nuclear deoxyribonucleic acid (DNA) [50] Around the end of the first trimester and start of the second trimester, toxic nicotine effects can cause abnormal remodeling of the spiral arteries supplying the placenta and reducing nutrition and oxygen transfer required for proper fetal growth. [50] Dysregulated functional and programming capacity of the placental-fetal unit is likely to influence preterm birth. [51–54]

Although, we have come close, we cannot establish this gestational window precisely because of the lack of detailed information in the dataset on when first trimester smokers quit and when second trimester initiators began smoking. Interestingly, smoking in all three trimesters was associated with a lower risk of preterm birth than smoking in the first two trimesters (referent), and the difference in risks was statistically significant. Because smoking is less likely to be overestimated in the first and second trimesters, we attributed these findings to a potential exposure misclassification error from suboptimal maternal self-reports of trimester of smoking on the birth certificate. [38, 48] In sensitivity analysis, under correct assumptions for binary exposure misclassification, [44–46] these findings were adjusted, raising three-trimester smoking risk to the level of two-trimester risk. Testing for effect modification by maternal race/ethnicity found no significant interaction which was consistent with prior research by Moore et al. [47]

To our knowledge, this is the first population-based study to examine how prenatal exposure measured by combined timing, duration and intensity of smoking is associated with an increased likelihood of delivering preterm. Ideally, the present study should be extended to reoccurring preterm birth and other adverse pregnancy outcomes. [55] Given the complexity of preterm birth syndrome, [56, 57] this epidemiological evidence integrated with biological and clinical knowledge will improve birth outcomes in women considered at a high risk of preterm delivery.

### Strengths and limitations

The main strength of our study was the availability of a large national dataset of over 2 million live U.S. births that contains information on maternal smoking behavior during each trimester of pregnancy. Revised birth certificates with enhanced health data on mothers and infants can expand opportunities in perinatal research. There are also a few limitations. One limitation is that the study population is not representative of the entire U.S. population, because in 2010 only 76% of all US states used the revised birth certificate. This still represents an advance over previous research that used data from one individual state [21, 47] or from a smaller set of states. [19] Similar to prior research, we excluded multiple births and data with missing covariates. There is a lack of information in the birth certificate on other risk factors that could confound the association between maternal smoking and preterm birth, such as exposure to passive smoking. Potential for exposure misclassification exists from maternal self-reports on smoking that may be underreported or prone to recall bias. [36, 41, 45] As we addressed in the sensitivity analysis, women who deliver preterm in the second trimester may inadvertently misreport smoking in the third trimester, becoming self-designated “third trimester smokers”.

## Conclusions

Women appear motivated to quit smoking during pregnancy; however, behavioral change is difficult to initiate and maintain. Knowing a period of vulnerability in which smoking exposure could harm a developing fetus and raise the risk of preterm birth would help health professionals to inform reproductive age and pregnant women. Early smoking cessation interventions may have a greater effect on the reduction of preterm birth, not just because the majority of women who smoke in the first trimester continue to smoke throughout pregnancy, but because the vulnerability period for smoking exposure appears late in the first trimester. Ultimately, findings from this study may guide future research in understanding the etiology and mechanisms of other adverse birth outcomes (e.g. low birthweight, placenta praevia) and in developing potential screening or risk prediction biomarkers and therapeutic targets.

## Abbreviations

aOR: Adjusted odds ratio
CI: Confidence interval
DNA: Deoxyribonucleic acid
IRB: Institutional Review Board
LMP: Last menstrual period
NCHS: National Center for Health Statistics
OE: Obstetric estimate
PRAMS: Pregnancy Risk Assessment Monitoring System
US: Ultrasound
